# The Influence of Anxiety on Postoperative Quality of Life Regarding Implant Treatments: An Epidemiological Study

**DOI:** 10.3390/dj12060165

**Published:** 2024-06-03

**Authors:** Carmen de la Calle Cañadas, Natalia Martínez-Rodríguez, Juan Santos-Marino, José María Martínez-González, Cristina Barona-Dorado

**Affiliations:** 1 Department of Conservative Dentistry and Prosthodontics, Complutense University of Madrid, 28040 Madrid, Spain; 2 Department of Clinical Dental Specialities, Complutense University of Madrid, 28040 Madrid, Spain; nataliamartinez@ucm.es (N.M.-R.); jmargo@ucm.es (J.M.M.-G.); 3 Department of Surgery, Faculty of Medicine, University of Salamanca, 37007 Salamanca, Spain; juansantos@usal.es

**Keywords:** dental anxiety, postoperative, dental implants

## Abstract

Dental treatment anxiety is highly prevalent worldwide. This is particularly important in the field of implantology since, in daily clinical practice, it translates into an increase in the difficulty of treatments, extending surgical times and having repercussions in the postoperative period. The aim of this multicentre, cross-sectional, epidemiological study was to determine the influence of anxiety levels in the postoperative period of an implant treatment on patients treated at two dental departments in Extremadura (Spain). To analyse anxiety levels, the modified Corah’s Dental Anxiety Scale questionnaire was administered before surgical treatment. To analyse the postoperative period, another questionnaire was provided 7 days after surgery. The study was conducted on a total of 102 patients (55 men and 47 women), with a mean age of 47.99 years. The results indicated that patients with a high and severe degree of anxiety had a poorer quality of life in general. Patients with a higher degree of anxiety perceived greater swelling at 24, 48, 72 h and one week after surgery.

## 1. Introduction

The concept of dental anxiety was originally defined by Corah et al. [1,2] as a patient’s response to the stress induced by visiting a dentist. Further definitions have emerged, such as the one proposed by Souza et al. [3], which states that it is a multisystemic response to a potential threat, constituting an individual and subjective experience that impacts daily life and is a major barrier to seeking dental care.

Anxiety scales have been developed to identify the degree of stress that patients experience when attending a dental appointment. For example, the Dental Fear Survey (DFS) was developed in 1973 purely for research purposes, the Dental Anxiety Scale (DAS) was published in 1978, and later on, the Modified Dental Anxiety Scale (MDAS) was developed, as the DAS had not included a question on local anaesthesia [1,2,3]. 

Dental anxiety is highly prevalent worldwide, affecting 5–42% of the population. Some studies report a range of 3–20% [4,5,6,7]. This wide range in prevalence corresponds to the fact that some studies provide figures for fear and others for anxiety [8]. Dental anxiety is a complex phenomenon that can be affected by different factors, such as age, gender, education, and nationality [9,10]. Most studies generally agree that it affects females more, and it has also been observed that young people exhibit higher levels of dental anxiety [3,9]. Some authors associate dental anxiety with a low socioeconomic status and/or poor education [8,11]; others, on the contrary, associate it with a higher level of education [12]. 

With regard to causal factors, memories of previous negative or painful experiences play an important role in the development of dental anxiety [5]. There are, however, studies that refer to patients experiencing dental anxiety who have never attended a dental appointment [5,9,13]. Family transmission has also been analysed as a causal factor. The parental pathway is considered to be one of the primary causes of dental fear acquisition in children [14]. It has also been associated with procedures requiring anaesthesia and/or the use of rotary instruments [5]. 

In the field of oral surgery and implantology, dental anxiety is particularly important since, in daily clinical practice, it results in an increase in the difficulty of administering treatments, especially if patients do not cooperate during their performance; this generates stress for the dental professional, increasing surgical times and having repercussions in the postoperative period [4,5,6,9,15,16]. Some of these repercussions are more pain and inflammation.

The main consequence of dental anxiety is a deficit in the oral health of a population due to the lack of regular check-ups or care [5,6]. Therefore, the challenge for practitioners is to understand and identify the early signs of dental anxiety in order to develop a rational treatment strategy that allows patients to comfortably visit their dentists and maintain the care needed to promote oral health. 

Due to the high prevalence of dental anxiety and its impact on oral health, it is vital to analyse the influence of this factor in the postoperative period of implant surgery.

The aim of this study was to determine the influence of anxiety on individuals’ postoperative quality of life after implant treatment among patients treated in two dental departments in Extremadura (Spain).

## 2. Method

### 2.1. Study Design

A cross-sectional, epidemiological study was designed following the recommendations of the STROBE (Strengthening the Reporting of Observational Studies in Epidemiology) initiative statement [17]. Approval was obtained from the Ethics and Clinical Research Committee of the Hospital Clínico San Carlos (Madrid) on 27 April 2020 (Code 20/281-E_TESIS).

### 2.2. Context

This study was carried out at two private dental centres in the Autonomous Community of Extremadura (Spain). The recruitment period was between September 2019 and January 2023, with a sample size of 102 patients.

### 2.3. Participants

The participants were adult patients who visited one of the participating centres with the intention of starting implantology treatment. The inclusion criteria were as follows: patients over 21 years of age who underwent implantology treatment and agreed to participate in the study after signing the informed consent form. The exclusion criteria were as follows: patients with an inability to complete the questionnaires, patients with abscesses or other acute infections that might cause oral sensitivity and surgeries including transalveolar maxillary sinus elevation, lateral maxillary sinus elevation and zygomatic implants, and patients for whom guided bone regeneration was necessary.

### 2.4. Variables

The variables studied included descriptive variables, implant placement surgery variables, and variables relating to dental anxiety and postoperative variables.

**Figure 1:**
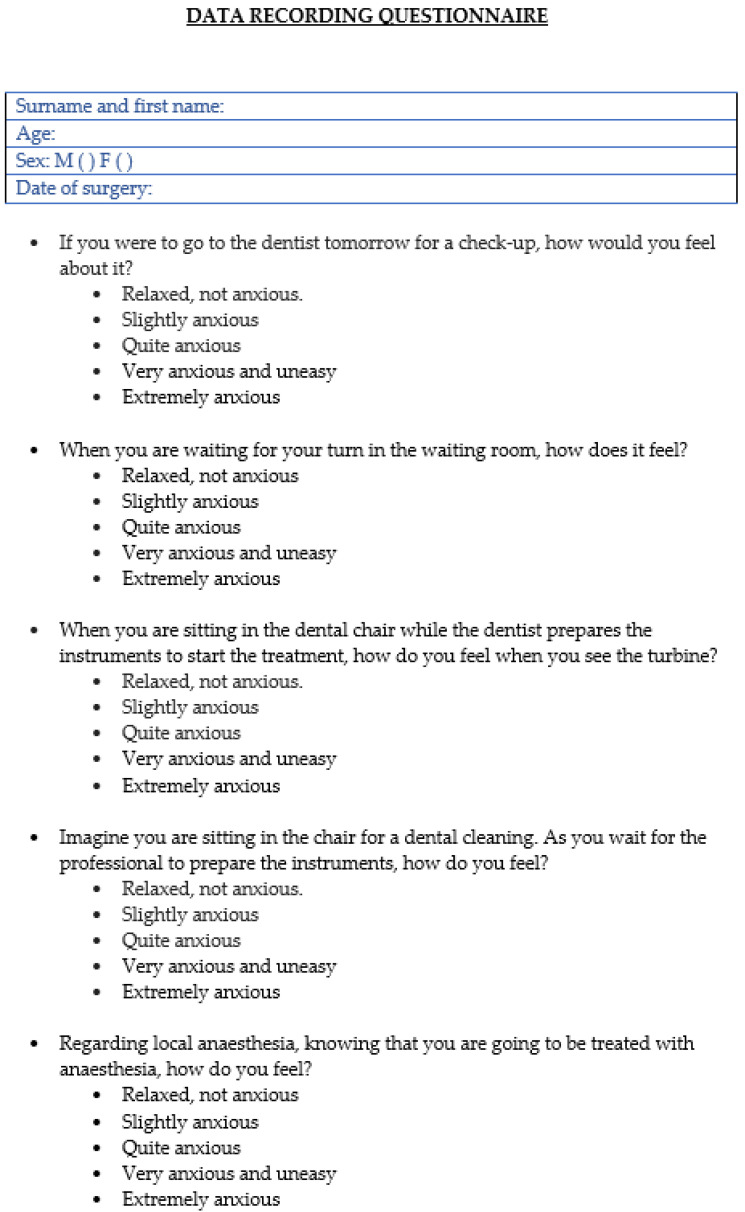
Modified Corah questionnaire.

Regarding the descriptive variables, age, sex, smoking habits, and the number of cigarettes/day in the case of an affirmative answer were recorded; the implant placement variables included the number of implants placed, the number of sites/quadrants involved in the surgery, and the surgical time. To assess the dental anxiety variables, the patients were administered the modified Corah anxiety questionnaire in paper form in Spanish [1] (Figure 1) before starting treatment at the dental office. The questionnaire includes five questions that refer to different factors that could create a stressful situation for patients. Responses are recorded on a 5-point Likert scale, ranging from ‘relaxed, not anxious’, scoring 1 point, to ‘extremely anxious’, scoring 5 points. The points for each answer are added up to establish the degree of anxiety, with <9 points indicating no or mild anxiety, 9–12 points indicating moderate anxiety, 13–14 points suggesting high anxiety, and >15 points representing severe anxiety levels.

The procedure was carried out under local anaesthesia, without premedication or sedation. After surgery, 750 mg of amoxicillin every 1/8 h for 7 days and 600 mg of ibuprofen every 1/8 h for 3 days were prescribed to all patients. Those allergic to penicillin were prescribed clindamycin at 300 mg for 7 days.

The postoperative anxiety variables were assessed using the postoperative questionnaire, which was administered 7 days after surgery; the following variables were recorded: whether the patient took sick leave; pain levels (on the day of surgery and at 24, 48, and 72 h, and the consumption of painkillers); the swelling perceived by the patient (on the day of surgery and at 24, 48, and 72 h); and quality of life (sick leave, work limitations, state of mind, weight loss, and fever).

### 2.5. Biases

Information bias was controlled by adapting the questions to the study population. When a patient came to the office with their dentist, the answers to each part of the questionnaire were reviewed together.

### 2.6. Sampling Size

The sample size calculation was carried out using the corresponding formula (*n* = k(Var1 + Var2)/(M1 − M2)^2^, where the mean difference (M1 − M2) is 1, the standard deviation is common in both groups (SD: 1.5), and the value corresponding to the 95% confidence level and 85% power is Zα + Zβ = 3.05. Therefore, n = 42. 

### 2.7. Statistical Methods

The statistical analysis was carried out at the Data Processing Centre of the Complutense University of Madrid, using the SPSS programme for Windows version 29.0. A descriptive analysis was carried out in which each of the qualitative and quantitative variables were reflected in a frequency table. After performing the Kolmogorov–Smirnoff and Shapiro–Wilk tests, it was concluded that the sample did not meet the criteria for normality. The statistical analyses were therefore carried out using the Mann–Whitney U test, the Kruskal–Wallis test, the Chi-squared test, and Kendall’s Rank Correlation Coefficient. The level of significance was set at *p* < 0.05, and all confidence intervals were given at the level of 95%.

## 3. Results

### 3.1. Participants

In total, 104 patients aged 21 or older were interviewed, and 102 were recruited, as 2 refused to sign consent forms for participation in the study. No loss occurred during the follow-ups.

### 3.2. Descriptive Data

Of the 102 patients, 55 were men (53.9%) and 47 were women (46.1%), with a mean age of 47.99 years. The minimum and maximum ages were 24 and 82, respectively. Of the total, 27 were smokers. The distribution of smokers was as follows: 1–10 cigarettes per day (59%, 16 patients); 10–20 cigarettes (19%, 5 patients); and >20 cigarettes (22%, 6 patients).

In terms of the implant placement variables, 37 patients received one implant (36.3%), 30 patients received two implants (29.4%), 17 patients received three implants (16.7%), 11 patients received four implants (10.8%), 4 patients received five implants (3.9%), and 3 patients received six implants (2.9%).

Regarding the number of quadrants, most patients underwent surgery in only one quadrant (55 patients, 53.9%), followed by two quadrants (43 patients, 42.2%), three quadrants (2 patients, 2%), and finally four quadrants (2 patients, 2%). The mean surgery time was 52.65 min, with a minimum duration of 25 min and a maximum of 120 min. 

When analysing the variables related to anxiety, it was found that 62 patients showed a mild degree of anxiety (60.8%), while 26 exhibited a moderate level of anxiety (25.5%), 7 showed high anxiety (6.9%) and another 7 showed severe anxiety levels (6.9%).

Table 1 shows the quality of life data obtained from the postoperative questionnaire, based on the following variables recorded 7 days after surgery: sick leave (one instance of sick leave was recorded), work limitations (six patients had work limitations, representing 5.9%), state of mind (seven had changes in the state of mind, amounting to 6.9%), weight loss (fifteen patients lost weight, corresponding to 14.7%), and fever (three had fever, constituting 2.9%). Table 2 shows the pain variable and its distribution.

Finally, the data for postoperative swelling are shown in Table 3.

### 3.3. Data on Result Variables

#### 3.3.1. Quality of Life

Patients with high and severe anxiety perceived a poorer quality of life after surgery (Fisher’s exact test *p* < 0.05). With regard to work limitations, weight loss, mood and fever, Fisher’s exact test revealed no differences between the different groups (*p* > 0.05).

#### 3.3.2. Pain

No association was observed between the degree of anxiety and pain on the day of surgery (Kruskal–Wallis, *p* = 0.185). An association was observed between the pain variables at 24, 48, 72 h and one week after surgery and a higher degree of anxiety (high and severe anxiety groups), (*p* < 0.05).

#### 3.3.3. Swelling

There was no relationship between anxiety and swelling on the day of surgery (Kruskal–Wallis, *p* = 0.275); however, among those patients with a higher degree of anxiety (high and severe anxiety groups), there was a perception of greater swelling 24, 48, 72 h and one week after surgery (Kruskal–Wallis *p* < 0.05).

#### 3.3.4. Age

The older the patient, the better their perception of quality of life after surgery (Kendall’s Rank Correlation Coefficient, *p* < 0.040). 

Pain was found to increase with age at 48 h (Kendall’s Rank Correlation Coefficient, *p* < 0.023). 

Finally, swelling was not affected by age (*p* > 0.05).

#### 3.3.5. Sex

Regarding the state of mind, 14.9% of women perceived changes, which can be compared to 0% for men (Fisher’s exact test *p* = 0.003). 

On the other hand, when analysing pain, no association with sex was found on the day of surgery or at 24, 48, and 72 h after surgery. However, there was an association during the week after surgery, as women perceived more pain than men (Mann–Whitney U test, *p* = 0.018). Women perceived greater swelling 24 h after implant placement (Mann–Whitney U test, *p* = 0.041), while men perceived greater swelling after 48 h (Mann–Whitney U, *p* = 0.006).

#### 3.3.6. Tobacco

When analysing the variables encompassing quality of life, no differences were observed between smokers and non-smokers. Forty-eight hours after surgery, smokers perceived more pain than non-smokers (Mann–Whitney U test, *p* = 0.008). Smokers did not perceive greater swelling on the day of surgery at 24 and 48 h but did perceive greater swelling at 72 h and one week later (*p* < 0.05). The number of cigarettes per day did not influence the smokers (*p* > 0.05).

#### 3.3.7. Number of Implants

The number of implants placed per patient did not influence the variables encompassing quality of life, pain, or perceived swelling (Kendall’s Rank Correlation Coefficient, *p* > 0.05). 

#### 3.3.8. Number of Locations and the Different Study Variables

When assessing the impact of the number of sites/quadrants operated on per patient, no differences were observed between the different groups (Fisher’s exact test, *p* > 0.05).

#### 3.3.9. Surgical Time and the Different Study Variables

Finally, there was also no association between the duration of the surgery and the variables quality of life, pain, or perceived swelling (Kendall’s Rank Correlation Coefficient, *p* > 0.05).

## 4. Discussion

### 4.1. Key Results

One of the most important results of the present study is that patients with high and severe degrees of anxiety perceived a poorer quality of life in general after surgery and more pain and swelling at 24, 48, and 72 h and in the following week. Therefore, it can be stated that a patient’s degree of dental anxiety influences the postoperative period after implant surgery.

The other variables considered, such as age, sex, smoking habits, the number of implants placed, the number of sites/quadrants operated on, and the duration of surgery, were evaluated separately, as it was considered that they could play a relevant role in the postoperative evaluation.

Firstly, it was observed that the older the patient, the better their perceived quality of life after surgery. In addition, as age increases, there is also an increase in pain at 48 h. Age is therefore a factor that needs to be taken into account when interpreting the results. It may seem contradictory that these patients perceived better quality of life but more pain at 48 h; however, there were no differences in pain at 24 and 72 h and one week later or in regard to swelling. Therefore, when considering the rest of the parameters, this is an isolated result.

In terms of gender, in the postoperative period, no alterations in the normal performance of work were observed among men and women. However, women showed more changes in mood. In addition, greater pain perception was observed in women in the week after surgery, along with greater swelling at 24 h (and at 48 h for men). Thus, it can be concluded that gender has an influence on the perception of quality of life, pain, and swelling in the postoperative period. 

Smokers perceived more pain than non-smokers 48 h after surgery and more swelling 72 h and one week later. Smoking therefore appears to have a negative influence on the postoperative period.

Finally, there were no differences in the postoperative period in terms of the number of implants, the quadrants operated on, or the duration of the surgery. This may be due to the fact that there were no major differences in the range of surgical times, as the minimum duration was 25 min and the maximum was 120 min.

### 4.2. Limitations

The main limitation of the present study is that, as it is a cross-sectional study, cause–effect inferences cannot be drawn [18]. Another drawback is that the study population visited their dentists with specific needs. Many of them came with more anxiety than usual since they knew they were about to undergo surgical treatment. Patients with higher levels of anxiety may not even be able to face attending the dentist at all. There was also a significant number of patients who had already been to other clinics to evaluate different treatment options and were already conditioned. 

With regard to the postoperative questionnaire, the possibility of using a questionnaire already published in the literature was considered; however, we did not identify one that referred to the three variables assessed (quality of life, pain, and swelling) in a complete manner, leading to the decision to individualise the questionnaire by adapting it to the objectives of the present study.

The patients analysed in this study may have certain common socio-demographic characteristics, thus limiting the extrapolation of data to populations from different social contexts; it would therefore be desirable to investigate other populations in future studies. 

### 4.3. Interpretation

Numerous factors are involved in the swelling process. Cytokines are released immediately after fibrin clot formation. Among them, the interleukin-1 (IL-1) family and prostaglandin E2 (PGE2) have been extensively studied due to their prominent activity and strong effects during the early stages of swelling, as well as the effects of physiological and psychological triggers on their secretion. Excessive concentrations of PGE2 and IL-1 have been assumed to cause an elevated inflammatory response. Psychological stress affects the inflammatory process by amplifying IL-1 production in the blood and saliva [19]. A recent publication addressed its influence on postoperative impacted mandibular third molars [20]. However, there are no publications linking dental anxiety to perceived swelling in the postoperative period after implants.

Khorshidi H et al. [21] analysed the influence of postoperative anxiety in 40 patients using questionnaires. The variables they analysed were bleeding, pain, difficulty eating and satisfaction. They found no differences. 

Fardal Ø et al. [22] conducted a study on 102 patients assessing the degree of anxiety and pain in the postoperative period after implant placement and periodontal surgery. In those patients with more anxiety, greater postoperative pain was perceived.

Zhang X et al. [23], in their study of 335 patients, conducted surveys with the same purpose, to analyse the influence of dental anxiety on pain perception during and after surgery; they concluded that anxious patients felt more pain in general.

Eli I et al. [24] conducted a study on 60 patients measuring anxiety and pain at different times and found a relationship. 

There are no published studies that look at quality of life, pain and swelling perception together.

Regarding differences between men and women concerning the swelling process, there have been many studies that show a sex difference in terms of wound healing; however, there is still no consensus on this [25]. Most researchers have assumed that sex hormones may play a role in this difference by regulating pro-inflammatory mediators; PGE2 is inhibited by testosterone and enhanced by oestradiol and progesterone [26,27].

Many studies have focused their efforts on assessing the influence of factors such as the number of implants, the quadrants operated on, and the duration of surgery on the postoperative period. This study, however, found no differences in the postoperative period when accounting for these factors. It has been recognised for many years that a patient’s emotions may be as crucial to the final outcome of a treatment as the technical aspects of oral surgery. Factors such as anxiety can influence a patient’s evaluation of a professional and a treatment [4,5,6,28]. This is reflected in the results of the present study, as it was found that the variables inherent to the surgery (number of implants, quadrants, and surgical time) did not influence the postoperative period.

## 5. Conclusions

Patients with high and severe dental anxiety perceive a poorer overall quality of life after surgery and more pain and swelling at 24, 48, and 72 h and one week after surgery.

The challenge for practitioners is to understand and identify the early signs of dental anxiety in order to develop a rational treatment strategy that allows patients to comfortably attend the practice and to maintain the necessary care in order to promote the importance of oral health. Therefore, future lines of study should aim to establish requirements for the appropriate management of this population group and thus lay the foundations for the way in which psychology and dentistry should work together for adequate oral health. 

## Figures and Tables

**Table 1:** Quality of life distribution: frequency and percentages.

Quality of Life	Response	Frequency	Percentage (%)
**Sick leave**	Yes	1	1
	No	101	99
**Limitations at work**	Yes	6	5.9
	No	96	94.1
**State of mind**	Yes	7	6.9
	No	95	93.1
**Weight loss**	Yes	15	14.7
	No	87	85.3
**Fever**	Yes	3	2.9
	No	99	2.9

**Table 2:** Pain distribution: frequency and percentages.

	Response	Frequency	Percentage
**Pain during day of surgery**	Pain-free	25	24.5
	Slight	41	40.2
	Moderate	24	23.5
	Intense	9	8.8
	Maximum	3	2.9
**Pain after 24 h**	Pain-free	34	33.3
	Slight	38	37.3
	Moderate	23	22.5
	Intense	5	4.9
	Maximum	2	2
**Pain after 48 h**	Pain-free	47	46.1
	Slight	40	39.2
	Moderate	9	8.8
	Intense	5	4.9
	Maximum	1	1
**Pain after 72 h**	Pain-free	71	69.6
	Slight	22	21.6
	Moderate	6	5.9
	Intense	2	2
	Maximum	1	1
**Pain the week after**	Pain-free	57	55.9
	Rarely	32	31.4
	Frequent	12	11.8
	Very frequent	1	1

**Table 3:** Swelling distribution: frequency and percentages.

	Response	Frequency	Percentage
**Swelling during day of surgery**	No swelling	38	37.3
	Slight	42	41.2
	Moderate	19	18.6
	Intense	3	2.9
**Swelling after 24 h**	No swelling	22	21.6
	Slight	41	40.2
	Moderate	32	31.4
	Intense	7	6.9
**Swelling after 48 h**	No swelling	36	35.3
	Slight	42	41.2
	Moderate	18	17.6
	Intense	6	5.9
**Swelling after 72 h**	No swelling	60	58.8
	Slight	29	28.4
	Moderate	10	9.8
	Intense	3	2.9
**Swelling the week after**	No swelling	86	84.3
	Slight	14	13.7
	Moderate	2	2

## Data Availability

The original contributions presented in the study are included in the article, further inquiries can be directed to the corresponding authors.
